# Transforming Global Health: Dr. Gagandeep Kang's Legacy in Microbiology and Public Health

**DOI:** 10.7759/cureus.70409

**Published:** 2024-09-28

**Authors:** Radha Kunjalwar, Akshunna Keerti

**Affiliations:** 1 Microbiology, Jawaharlal Nehru Medical College, Datta Meghe Institute of Higher Education and Research, Wardha, IND; 2 Internal Medicine, Jawaharlal Nehru Medical College, Datta Meghe Institute of Higher Education and Research, Wardha, IND

**Keywords:** biographies, historical vignette, historical vignettes, medical innovation, medical stories, public health, rotavac, vaccination

## Abstract

Dr. Gagandeep Kang is a towering personality in the field of microbiology and is known for her groundbreaking work globally. She made seminal discoveries in areas of enteric infections, vaccine development, and public health that have created a sea of change in scientific understanding and healthcare, probably beyond the borders of India. It was in honor of her achievements in science and the continuing impact of her studies on microbiology and public health that this review article was undertaken. The purpose of the article, therefore, was to incorporate a detailed analysis of her work primarily for an advanced and professional audience of microbiologists.

## Introduction and background

Dr. Gagandeep Kang is a luminary in the area of microbiology whose inter-disciplinary research work on the development, transmission, and prevention of enteric infections has left a lasting impact. She has taken over the pioneering lead in the study of enteric diseases and the development of vaccines, especially the typhoid vaccine [1]. Microorganisms that cause intestinal illnesses, including bacteria, viruses, and parasites, are the cause of enteric diseases. The most common cause of these illnesses is ingestion of contaminated food or water. This includes a variety of illnesses such as salmonellosis, shigellosis, typhoid fever and many more [2]. Dr. Kang has certainly reinforced herself as a leader whose research is truly pragmatic and far-reaching in the general area of global health. Her career testifies to the power of hard scientific inquiry underpinned by a deep commitment to betterment in public health outcomes, especially in low- and middle-income countries. Worldwide, rotaviruses are the cause of acute viral gastroenteritis. The variety of rotaviruses identified in India in the 1980s and 1990s highlights the necessity of cocirculating strain surveillance to track swift changes in circulation and identify new strains. In this review article, we will discuss the great life of Dr. Kang and her epic works in scientific conquests, all the prominent awards she has received, and the contributions she has made to the field of microbiology [2,3]. We will take a walk through her illustrious career and look into the work that has stamped its mark on the field and has made significant contributions to the betterment of society.

## Review

Early life and education

Dr. Gagandeep Kang was born in 1962 in Vellore, Tamil Nadu, India. She completed her schooling in the field of science. After obtaining a degree in medicine from Christian Medical College (CMC), Vellore, one of the leading medical institutions in the country, Dr. Kang chose microbiology as her further specialization. She was recognized as academically strong from a very early age and quickly proved to be a very promising researcher. Her postgraduate training in microbiology was completed at CMC, under the guidance of stalwarts in microbiology. She became a researcher of infectious diseases and the field of epidemiology of enteric infections later became the cornerstone of her research career [2].

Career and major contributions

Vaccine Production and Enteric Diseases

Among Dr. Kang's most exceptional research works in microbiology is the study of enteric diseases, in particular, the diseases caused by rotavirus, the source of extreme diarrhea among children globally. Her research work has been termed groundbreaking since the information has been vital for figuring out rotavirus epidemiology and the development of vaccines for rotavirus. Dr. Kang was in the thick of formulating the first indigenous rotavirus vaccine in India, ROTAVAC. This public-private partnership led to antigen development and the consequent vaccine, significantly impacting rotavirus disease in low- and middle-income countries like India. Her work was imperative in convincing the governments to include ROTAVAC in their national immunization program. The work on the rotavirus vaccine that she has carried out has potentially saved millions of lives and has set the stage for other vaccines to be developed in the region. Her research on rotavirus vaccines is a testament to the fact that the vaccines, even from low- and middle-income countries, actually work as good as those from high-income countries, based on rigorous science with clinical trials [3].

Public Health Impact

The impact of Dr. Kang's work in public health goes far beyond vaccine development. Her work on enteric infections has had a major impact on the public health policy in India and other developing countries. She advocated extensively for including vaccines in national immunization programs, and her investigations provided the necessary supportive evidence, with her studies on cholera, typhoid, Shigella and other enteric pathogens against rotavirus [4]. The foundation of several interventions for these diseases lies in the work of Dr. Kang, who has been able to give the required evidence in the field of interventions for strategies of control and prevention of these diseases in settings where they are endemic. Dr. Kang has also worked on the issue of enteric infections to emphasize the significance of basic sanitation and hygiene in their prevention. This has been one of the main areas fueling her strong advocacy for improved Water, Sanitation and Hygiene (WASH) in low- and middle-income countries to reduce the burden of these diseases. It has been shown that interventions could be more effective and synergistic if applied in synergy with vaccination to reduce the incidence burden of these diseases [5].

Leadership in Science and Academia

Along with her main occupation as a researcher, she has been in charge of several critical leadership roles in academia and science. She was the executive director (2016-2020) of the Translational Health Science and Technology Institute (THSTI), an autonomous institute under the Department of Biotechnology, Government of India, playing a vital role in initializing the country's translational research and innovation programs [5]. Under her leadership, THSTI grew into a hub of cutting-edge research in infectious diseases, maternal and child health, and other critical areas of public health. Dr. Kang has also been serving on various international committees and advisory boards spread globally, including that of the World Health Organization (WHO), influencing global health policy dialogues and decision-making processes. With her vast knowledge of vaccines and public health research, she is called upon for advice on infectious diseases and global health [6].

Recognition and Awards

**Figure 1:**
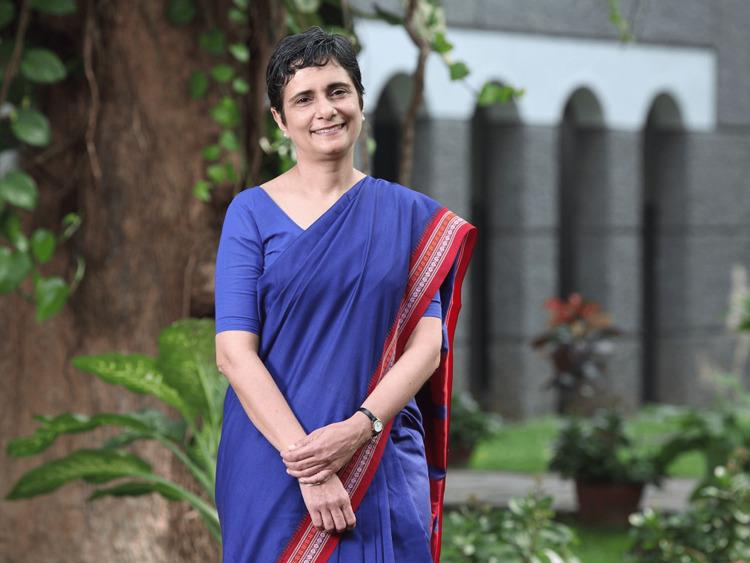
Dr. Gagandeep Kang Prof. Gagandeep Kang was awarded the Infosys Prize in life sciences for her breakthrough contributions to understanding the natural history of rotavirus and other infectious diseases that are important both on national and international levels [9]. Image taken from an open source.

Numerous awards and honours also supplement her work in science and public health. She became the first Indian woman to be elected as a Fellow of the Royal Society in London in 2019, which was indeed an international recognition for her breakthrough work in the field of microbiology and public health not only in India but globally as well. Besides the fellowship of the Royal Society, other influential honours include the Infosys Prize in Life Sciences, the Dr. C. G. Pandit National Chair of the Indian Council of Medical Research, and the Dr. B. C. Roy Award for great contributions toward medical research [7]. Her extensive cohort-based epidemiological, environmental, and clinical trial research on enteric diseases in children and their effects on life course has recently earned her the prestigious John Dirks Canada Gairdner Global Health Award, 2024. Her research has significantly impacted vaccine development and health policy in India and around the globe. She led research projects that helped create and implement two Indian rotavirus vaccines into the country's immunization program [8-10]. Dr. Kang has received numerous accolades and prizes from the Government of India for her groundbreaking work. Among these are the Government of India's 2006 Woman Bioscientist of the Year award. She also got elected to fellowship in the American Academy of Microbiology (2010), Indian Academy of Sciences (2011), National Academy of Sciences (2013), Faculty of Public Health in the United Kingdom (2015) and Indian National Science Academy (2016). Serving on multiple national and international editorial boards, research-funding review boards, and scientific advisory committees, she has played a trailblazing role in the field of biomedical sciences (Figure 1) [9].

Mentorship and advocacy

Dr. Kang's impact on microbiology extends beyond her work and leadership. She has been an outstanding mentor and role model to young scientists and researchers, especially women. Her successful career milestones and further dedicated commitment to public health have inspired all [11]. Dr. Kang has been a great champion of the cause of increased participation of women in science. Her mentorship has been impactful especially in India, where she has helped build a new generation of scientists committed to working on some of the country's most critical challenges in public health. This ranges from her efforts in promoting diversity in science to her being an effective lobbyist for making the scientific community just and open, showing quite obviously Dr. Kang's commitment to mentoring and advocacy [12]. Dr. Gagandeep Kang has spent the last 30 years working in the field of enteric illness research, giving her a broad view of many of the health issues her home country India is facing. Research on typhoid disease is another crucial field for her. She has declared that her juniors will carry forward her study on the typhoid vaccine, and she will be the adjunct investigator. In addition to serving in several national and state advisory capacities in India when COVID-19 emerged, she was a key member of the WHO's South-East Asia Regional Immunization Technical Advisory Group and the Strategic Advisory Group of Experts on Immunization (SAGE), on COVID-19 vaccines. Dr. Kang's will to influence a health system change is strengthened by the effect of COVID-19 on growing health disparities in India [13].

Discussion

Dr. Kang's research on enteric diseases has also significantly impacted global health. Her insights into the epidemiology and transmission of enteric diseases now constitute the basis for public health approaches toward their control and prevention. Furthermore, she has been responsible for causing immense positive influence on the public health policies of India and other countries by reiterating, through advocacy, strategies like sanitation, hygiene, and vaccination in controlling and reducing diseases [13]. In addition, she has led from the front scientific and academic pursuits, thereby contributing a big fillip to translational research and innovation in India. Her contributions to institution building, both for research and research organizations, building leadership skills in younger scientists, and driving diversity and inclusion in science have been very impactful in the scientific community, not only in India but the world over as well. Her election to Fellowship of the Royal Society and the numerous other awards that she has received are a testimony of her contributions to microbiology and public health par excellence [13]. Her work is unique as she stands apart based on the scientific rigour of her work and more importantly, its practical impact. The direct health-related implications are for millions, particularly for developing countries in the low- to middle-income brackets where the burden of infectious diseases is the greatest. The development of the ROTAVAC vaccine was a milestone achievement that saved thousands of lives and set new benchmarks for vaccine development in the region [14]. As not a lot of research work has been done on Dr. Kang's study subjects, not a lot of information is available. This might be considered a limitation of this article. This review is totally based on her research on enteric diseases and her role in vaccine development. More research work should be done to illustrate her national as well as international work on vaccine development and recent advances.

## Conclusions

The illustrious career of Dr. Gagandeep Kang is a testimony of how stern scientific research and a relentless desire to affect the community and public health go hand in hand. Her study of enteric diseases and vaccine development has great implications, providing the advancement of scientific knowledge and an impactful effect on improving health outcomes for millions of people. She remains committed to her work in scientific research and public health, academics, and mentoring. As we reflect on Dr. Kang's contributions to microbiology and public health, it is clear that her work has left an indelible mark on the field. Her achievements are a testament to the role of scientific research in fighting the most crucial health challenges of our time. Presently, Dr. Kang is a preeminent investigator specialising in viral infections, and serves on numerous committees that facilitate the formulation of national health policies. Dr. Kang's legacy will continue to inspire and influence future generations of scientists and public health professionals.
